# Characterization of highly active 2-keto-3-deoxy-L-arabinonate and 2-keto-3-deoxy-D-xylonate dehydratases in terms of the biotransformation of hemicellulose sugars to chemicals

**DOI:** 10.1007/s00253-020-10742-5

**Published:** 2020-06-21

**Authors:** Samuel Sutiono, Bettina Siebers, Volker Sieber

**Affiliations:** 1 Chair of Chemistry of Biogenic Resources, Campus Straubing for Biotechnology and Sustainability, Technical University of Munich, Schulgasse 16, 94315 Straubing, Germany; 2 Molecular Enzyme Technology and Biochemistry (MEB), Environmental Microbiology and Biotechnology (EMB), Centre for Water and Environmental Research (CWE), University of Duisburg-Essen, Universitätsstraße 5, 45117 Essen, Germany; 3 Catalytic Research Center, Technical University of Munich, Ernst-Otto-Fischer-Straße 1, 85748 Garching, Germany; 4 Straubing Branch BioCat, Fraunhofer IGB, Schulgasse 11a, 94315 Straubing, Germany; 5 School of Chemistry and Molecular Biosciences, The University of Queensland, 68 Copper Road, St. Lucia, 4072 Australia

**Keywords:** L-Arabinose, D-Xylose, Dehydratase, Weimberg, Biotransformation, Chemicals

## Abstract

2-keto-3-L-arabinonate dehydratase (L-KdpD) and 2-keto-3-D-xylonate dehydratase (D-KdpD) are the third enzymes in the Weimberg pathway catalyzing the dehydration of respective 2-keto-3-deoxy sugar acids (KDP) to α-ketoglutaric semialdehyde (KGSA). The Weimberg pathway has been explored recently with respect to the synthesis of chemicals from L-arabinose and D-xylose. However, only limited work has been done toward characterizing these two enzymes. In this work, several new L-KdpDs and D-KdpDs were cloned and heterologously expressed in *Escherichia coli*. Following kinetic characterizations and kinetic stability studies, the L-KdpD from *Cupriavidus necator* (*Cn*L-KdpD) and D-KdpD from *Pseudomonas putida* (*Pp*D-KdpD) appeared to be the most promising variants from each enzyme class. Magnesium had no effect on *Cn*L-KdpD, whereas increased activity and stability were observed for *Pp*D-KdpD in the presence of Mg^2+^. Furthermore, *Cn*L-KdpD was not inhibited in the presence of L-arabinose and L-arabinonate, whereas *Pp*D-KdpD was inhibited with D-xylonate (I_50_ of 75 mM), but not with D-xylose. Both enzymes were shown to be highly active in the one-step conversions of L-KDP and D-KDP. *Cn*L-KdpD converted > 95% of 500 mM L-KDP to KGSA in the first 2 h while *Pp*D-KdpD converted > 90% of 500 mM D-KDP after 4 h. Both enzymes in combination were able to convert 83% of a racemic mixture of D,L-KDP (500 mM) after 4 h, with both enzymes being specific toward the respective stereoisomer.

**Table:** 

**Key points** *• L-KdpDs and D-KdpDs are specific toward L- and D-KDP, respectively.* *• Mg*^*2+*^ *affected activity and stabilities of D-KdpDs, but not of L-KdpDs.* *• CnL-KdpD and PpD-KdpD converted 0.5 M of each KDP isomer reaching 95 and 90% yield.* *• Both enzymes in combination converted 0.5 M racemic D,L-KDP reaching 83% yield.*

**Electronic supplementary material:**

The online version of this article (10.1007/s00253-020-10742-5) contains supplementary material, which is available to authorized users.

## Introduction

Petrochemicals have been utilized by humans for almost a century in producing chemicals and other building blocks that are used in everyday life. The utilization of fossil resources, which are the primary source of petrochemicals, has long been a concern as regards sustainability and climate change. The use of plant-derived raw materials has been proposed in order to alleviate our dependency on petrochemicals. In the first generation, starchy materials were utilized, mainly for bioethanol fermentation and lactic acid production (Naik et al. 2010; Mohr and Raman 2015). This approach, while being more environmentally friendly, is still under debate, in particular in relation to competition with food sources (Naik et al. 2010; Mohr and Raman 2015; Rulli et al. 2016). The second-generation bio-production of fuel and chemicals has been developed in order to avoid the potential of competition. In this approach, lignocellulosic biomass is utilized. With annual production of dried biomass exceeding 220 billion tons, it provides us with an enormous amount of truly renewable raw materials (Huang and Fu 2013; Isikgor and Becer 2015).

Unlike the first-generation raw materials, lignocellulosic biomass consists of the following three different components: cellulose, hemicellulose, and lignin. Cellulose can be hydrolyzed to yield D-glucose. Fermentation of D-glucose to chemicals is a straightforward process because it is a universal substrate for most industrially relevant microorganisms. During degradation of the second carbohydrate polymer, hemicellulose will result in several pentose and hexose sugars, of which D-xylose and L-arabinose are two major constituents (Isikgor and Becer 2015). In contrast to D-glucose, these hemicellulose sugars are not easily fermentable. Several works have thus been focused on introducing pentose metabolism pathways in industrially relevant hosts in order to establish hexose and pentose co-fermentation and, therefore, to increase the feasibility of biomass utilization (Fernandes and Murray 2010; Chandel et al. 2011).

There are three major pathways found in nature that metabolize D-xylose and L-arabinose. The first pathway is the pentose phosphate pathway, which is accessed after isomerization of the pentose sugars from the aldolase to the ketose form. The second and third pathways are called non-phosphorylated oxidative pathways (Jagtap and Rao 2018; Valdehuesa et al. 2018). In the latter pathways, pentose sugars are oxidized to their respective sugar acids. Dehydration at position C2 and C3 results in 2-keto-3-deoxy-D-xylonate and 2-keto-3-deoxy-L-arabinonate, respectively. At this point, the two pathways are branched. In one pathway, aldolase will split the respective intermediates to pyruvate and glycoaldehyde. This pathway is known as the Dahms pathway (Stephen Dahms 1974). The alternative takes place via further dehydration of the 2-keto-3-deoxy-D-xylonate and 2-keto-3-deoxy-L-arabinonate at positions C4 and C5 by means of a dehydratase producing α-ketoglurate semialdehyde (KGSA). Oxidation of the terminal aldehyde of KGSA will yield α-ketoglutarate (α-KG), which is an intermediate of the TCA cycle. This pathway is known as the Weimberg pathway (Weimberg 1961). Because the intermediates at the branch point (2-keto-3-deoxy-D-xylonate and 2-keto-3-deoxy-L-arabinonate) lose their chirality at C2 and C3, all D-pentose sugars (D-xylose, D-ribose, D-arabinose, and D-lyxose) will result in the same intermediate after undergoing one oxidation followed up by the subsequent dehydration at C2 and C3. This is also true for their respective L-sugars. D and L functionality is maintained because it is dictated by the position of a hydroxyl group at C4. Therefore, 2-keto-3-deoxy-D-xylonate and 2-keto-3-deoxy-L-arabinonate are referred to herein as 2-keto-3-deoxy-D-pentonate (D-KDP) and 2-keto-3-deoxy-L-pentonate (L-KDP), respectively, as used in the nomenclature from a previous study (Watanabe et al. 2006). The enzymes that catalyze the dehydration reactions are thus called D-KdpD and L-KdpD, respectively.

**Fig. 1:**
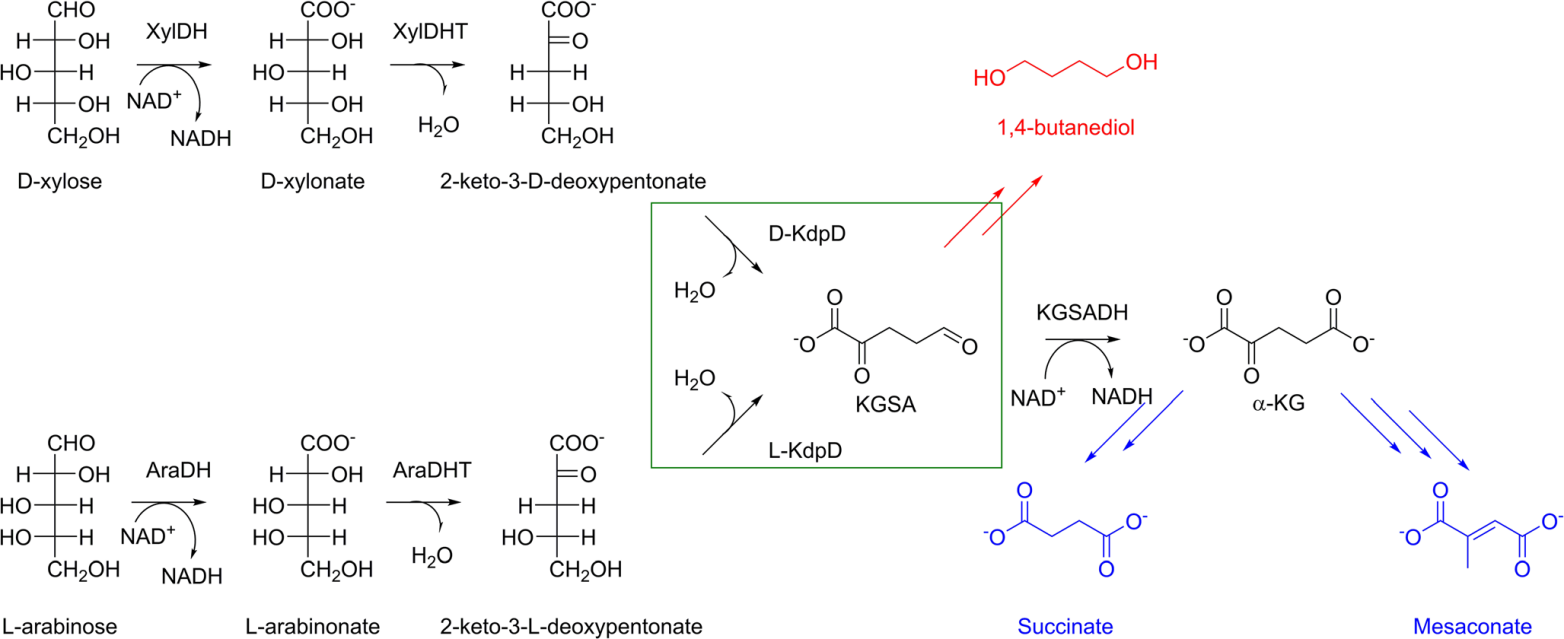
The Weimberg pathway and its subsequent modification for the conversion of L-arabinose and D-xylose to added-value compounds/chemicals. The natural pathway is shown in black. The conversions catalyzed by L-KdpD and D-KdpD that are the focus of this study are highlighted by a green box. The pathways are adapted from previous studies (Bai et al. 2016; Tai et al. 2016)

Introduction of the Weimberg pathway in industrially relevant microorganisms will allow the production of α-KG and its subsequent derivatives in the TCA cycles from D-xylose and L-arabinose (Tai et al. 2016; McClintock et al. 2017). A-KG is a promising building block for the synthesis of heterocyclic compounds (Stottmeister et al. 2005). A-KG has also widely been used in animal feed and for humans as a food and medicine additive (Wu et al. 2016; Liu et al. 2018). The longevity effect of α-KG in *Caenorhabditis elegans* was also recently shown (Chin et al. 2014)*.* The Weimberg pathway has also been explored further in recent years in order to determine its potential toward the synthesis of non-natural chemicals from the hemicellulose sugars. Several platform chemicals that have been produced from modified Weimberg pathways include 1,4-butanediol (BDO), mesaconate, glutamate, and succinate (Fig. 1) (Bai et al. 2016; Tai et al. 2016; McClintock et al. 2017). These are important platform chemicals for the production of several polymers. The dehydration of BDO results in tetrahydrofuran, which is a building block for Spandex and an industrial solvent. Decarboxylation of mesaconate will yield methacrylic acid, which is a precursor of vinyl ester resin (Yadav et al. 2018). Succinate has been utilized in the production of polyester, surfactants, detergent, food additives, and pharmaceuticals (Saxena et al. 2016).

In order to design efficient pathways in a microorganism, the kinetic characterization of responsible enzymes is indispensable. This parameter is equally important when an in vitro approach is preferred. In vitro approaches to producing chemicals have gained increasing attention in recent years as an alternative to in vivo (Guterl and Sieber 2013; Dudley et al. 2015; Claassens et al. 2019). In contrast to the other enzymes of the Weimberg pathway, D-KdpD and L-KdpD have only been characterized partially. It has been reported that *Caulobacter crescentus* and *Paraburkholderia xenovorans* are able to encode functional D-KdpD. However, their activities are rather low (k_cat_: 0.5 s^−1^ and K_M_: 1.9 mM for *Cc*D-KdpD; k_cat_: 4.7 s^−1^ and K_M_: 9 mM for *Px*D-KdpD) (Tai et al. 2016). With regard to *Cc*D-KdpD in particular, its activity seems to be much lower than the two preceding enzymes of the Weimberg pathway in *C. crescentus* (D-xylose dehydrogenase and D-xylonate dehydratase) (Andberg et al. 2016). Regarding L-KdpD, one elegant work from the 1960s previously characterized a functional enzyme from *Pseudomonas saccharopila* (also known as *Pelomonas saccharophila*) (Stoolmiller and Abeles 1966). Possible catalytic mechanisms have also been proposed on the basis of isotopes and inhibitor studies (Portsmouth et al. 1967). However, no protein sequence is available, which one would be necessary for easy heterologous protein expression. The protein sequence of a homologous enzyme from *Azospirillum brasilense* (*Ab*L-KdpD) has been reported recently and exhibits an activity of 20 U/mg (k_cat_ ~ 11 s^−1^) (Watanabe et al. 2006). Another homologous enzyme from *Bukholderia multivorans* (*Bm*L-KdpD) has also been reported, but it exhibits considerably lower activity (k_cat_ of 0.2 s^−1^) (Tai et al. 2016).

Therefore, several D-KdpDs and L-KdpDs have been characterized in detail in this study. *Cc*D-KdpD and *Ab*L-KdpD were used as guide sequences to mine homologous enzymes. A chemoenzymatic approach was applied to synthesize the substrates D-KDP and L-KDP from D-xylose and L-arabinose, respectively. In addition to kinetic parameters, pH optimum, temperature stability, and inhibitory effects of substrates and intermediates in the Weimberg pathway were determined. The best enzyme variants with respect to activity and stability were applied in the conversion of high substrate load (> 0.5 M).

## Materials and methods

### Cloning, expression, and enzyme purification

*Caulobacter crescentus* (DSM 4727), *Pseudomonas putida* KT 2440 (DSM 6125), and *Cupriavidus necator* H16 (DSM 428) were purchased from DSMZ (Germany). All cells were grown according to the protocol described by DSMZ for isolating their gDNA. The isolation of gDNA was performed using the DNeasy UltraClean Microbial Kit (Qiagen, Germany). gDNA of *Herbaspirillum seropedicae* Z67 (DSM 6445) was purchased directly from DSMZ. D-kdpD and L-kdpD genes were amplified from the corresponding gDNAs using a two-step PCR protocol (PCR without annealing step). In brief, the PCR mix consisted of the following: Phusion high-fidelity polymerase 1 U (NEB, Germany), 2 mM of dNTPs (VWR; Germany), 0.5 mM of forward and reverse primers, 25 ng of gDNA, 1x GC buffer (NEB, Germany), and ddH_2_O up to 50 μL. The PCR reactions were as follows: 98 °C for 30 s, 30 cycles at 98 °C for 10 s, 72 °C for 1 min, and 72 °C for 5 min, and an indefinite hold at 16 °C. Upon PCR, the products obtained were separated using agarose gel electrophoresis. Bands with correct sizes were excised and purified using the NucleoSpin Gel and PCR Clean up kit (Machery and Nagel, Germany). Purified DNAs were then digested and ligated into either pET28a or pET24a (Invitrogen, Germany) to give corresponding N- or C-terminal hexa-histidine tags. Another kdpD1 gene from *Caulobacter crescentus* (*Cc*D-kdpD1), D-kdpD gene from *Paraburkholderia xenovorans* (*Px*D-kdpD), as well as L-kdpD genes from *Azospirillum brasilense* (*Ab*L-kdpD) were ordered from ATG Biosynthesis (Germany) as optimized codons for expression in *E. coli*. The *Cc*DkdpD1 gene was synthesized according to the sequence described in the previous study (Tai et al. 2016). These genes were also cloned to either pET28a or pET24a using appropriate restriction enzymes. The ligated plasmids were used to transform *E. coli* BL21 (DE3) (Invitrogen, Germany). Forward and reverse primers used to amplify the genes as well as restriction enzymes used for cloning to pET vectors are presented in Table S1 in the Supporting Information.

To express the enzymes, a single colony of *E. coli* BL21 (DE3) possessing the correct gene was grown overnight in 10 mL LB medium supplemented with 100 μg/mL kanamycin in a 100 mL baffled flask at 37 °C, 150 rpm. The overnight culture was then transferred to a 2 L baffled flask containing 500 mL autoinduction media supplemented with 100 μg/mL kanamycin (Studier 2005). The culture was incubated at 30 °C, 120 rpm for 16 h. The cells were pelleted by centrifugation at 4000 ×*g* for 15 min. After decanting the supernatant, the cell pellet was transferred to a 50 mL falcon tube for subsequent purification.

A binding buffer (50 mM KPi pH 8, 20 mM imidazole, 500 mM NaCl, and 10 vol% glycerin) was added up to 40 mL in the 50 mL falcon tube. A total of 5 μg/mL DNase (AppliChem) and 2 mM MgCl_2_ were added to the cell suspension. Cells were disrupted by sonication (80% and cycle 0.5 s) in ice for 20 min. The solution was then cleared by centrifugation at 20,000 ×*g* for 30 min. The supernatant was filtered through a 0.45 μm cellulose filter (VWR, Germany) before application to an Äkta purifier (GE Healthcare, Germany). A 5 mL HisTrap FF Crude (GE Healthcare, Germany) was used for purification of His-tagged proteins. The column was washed with 30 mL binding buffer to remove *E. coli* proteins. An elution buffer (50 mM KPi, pH 8, 500 mM imidazole, 500 mM NaCl, and 10 vol% glycerin) was used to elute the His-tagged protein from the column. The buffer was exchanged using a HiPrep desalting column (GE Healthcare, Germany). A total of 50 mM HEPES pH 7.5 was used as the final buffer for all enzymes in this study. After the buffer exchange, all enzymes were flash-frozen in liquid nitrogen and stored at − 80 °C until further use. The purity and size of the enzymes were analyzed via SDS-PAGE.

#### Synthesis of 2-keto-3-deoxy-L-pentonate and 2-keto-3-deoxy-D-pentonate

A chemoenzymatic approach was applied for synthesizing the substrates (L-KDP and D-KDP) from their respective aldoses. This approach was modified from previously published work (Sperl et al. 2016). L-Arabinose and D-xylose were oxidized separately using a 0.5% gold catalyst (Evonik, Germany) in the presence of saturated oxygen. Oxidation was performed in an automatic titrator (SI Analytics, Germany). The pH and temperature were set to 8 and 50 °C, respectively. The gold catalyst was used at a ratio of 0.033 g/g sugar. The oxygen flow rate used was 40 mL/min, and the reaction was mixed using the magnetic stirrer of the automatic titrator. The oxidation was started by the addition of the gold catalyst. After the oxidation was finished (yield > 97% for L-arabinose and D-xylose), the catalyst was pelleted by centrifugation at 4000 ×*g* for 15 min. The sugar acid (L-arabinonate and D-xylonate) solutions were filtered through a 0.45 μm cellulose filter.

Dehydration of the sugar acids was performed as follows. *Cc*XylDHT was used to dehydrate D-xylonate and *Rl*AraDHT to dehydrate L-arabinonate (Andberg et al. 2016). The expression of each enzyme was performed as described previously (Sutiono et al. 2020). Dehydration of 37.5 mmol of the sugar acids was achieved by using 5 mg of each enzyme. Magnesium chloride (5 mM) was added to the reaction as a cofactor for both enzymes. No buffer was used in the reaction. After an overnight reaction, > 99% conversion of D-xylonate and L-arabinonate was achieved. Given that this was an HPLC analysis and no sugar acid or other peaks were observed beyond a single product peak, it was assumed that > 99% conversion of the sugar acids resulted only in 2-keto-3-deoxy-D-pentonate and 2-keto-3-deoxy-L-pentonate. The final product of the dehydration reaction was a clear solution for both KDPs. The solutions were filtered using an Amicon Ultra Centrifugal Filters 10K (Sigma Aldrich, Germany) to remove the enzymes. The solutions were stored at − 20 °C until further use. No apparent substrate decomposition after being stored > 3 months was observed by HPLC. The standard HPLC method to detect sugar acids and their subsequent dehydrated product was described in earlier research (Guterl et al. 2012).

#### Kinetic characterization of the dehydratases

To determine dehydratase activity, a coupled-assay with *Pp*KGSADH as auxiliary enzyme was used to link the oxidation of KGSA to α-KG using NAD^+^, as was described in a previous study (Beer et al. 2017). The measurement was performed in a 96-well F-bottom plate (Greiner Bio-One, Germany). 20 μL of a diluted enzyme was added to a 96-well plate. A total of 180 μL of reaction solution containing different concentrations of either D-KDP or L-KDP with 0.5 U/mL *Pp*KGSADH, 2 mM NAD^+^, 5 mM MgCl_2_, 50 mM HEPES pH 7.5 (end concentration) were added subsequently. The measurement was performed with a Multiskan spectrophotometer (Thermo Scientific, Germany) by measuring the development of NADH at 340 nm at 25 °C. Enzyme activity (k_cat_) was defined as the number of NADH molecules formed per molecule of enzyme per second. Measurements were performed in triplicate.

#### Effect of Mg^2+^ on activity, kinetic, and thermodynamic stability

Two different enzyme stock solutions were used to determine activities. The first stock was a standard enzyme solution, whereas the second stock was an enzyme solution that had been pre-incubated with 5 mM EDTA at 25 °C for 1 h. A total of 20 μL of a diluted enzyme solution from the first stock was added to a 96-well F-bottom plate. A total of 180 μl of reaction mix containing 5 mM of each substrate (L-KDP for *Cn*L-KdpD and *Hs*L-KdpD; D-KDP for *Pp*D-KdpD and *Hs*D-KdpD), 0.5 U/mL *Pp*KGSADH, 2 mM NAD^+^, and 50 mM HEPES pH 7.5, without and with 5 mM MgCl_2_ was added. The development of NADH was monitored at 340 nm using Multiskan spectrophotometry. The same reaction mixture without MgCl_2_ was also used to measure activity of the second enzyme stock. All measurements were performed at 25 °C.

To measure T_50_^1h^ (temperature at which an enzyme loses 50% of its initial activity after 1 h incubation), 20 μL of enzyme solution was transferred to a 96-PCR plate (Brand, Germany). The enzyme solution contained 1 mg/mL enzyme in 50 mM HEPES pH 7.5 with 5 mM EDTA, with or without 5 mM MgCl_2_. The PCR plate was incubated in a thermal cycler (MyCycler, Bio-Rad, Germany) at gradient temperatures from 30 to 55 °C. The incubation was performed for 1 h. Following this heating step, the enzyme solution was diluted accordingly with 50 mM HEPES pH 7.5. A total of 20 μL of diluted solution was transferred to a 96-well plate. A total of 180 μL of reaction solution containing 5 mM of each substrate (L-KDP for *Cn*L-KdpD and *Hs*L-KdpD and D-KDP for *Pp*D-KdpD and *Hs*D-KdpD), 0.5 U/mL *Pp*KGSADH, 2 mM NAD^+^, 2.5 mM MgCl_2_, 50 mM HEPES pH 7.5 (end concentration) was added. The development of NADH was monitored at 340 nm using a Multiskan spectrophotometer. The activities obtained were normalized to the highest value. Three independent repeats were performed.

The thermodynamic stability is displayed as the melting temperature (T_m_) and was analyzed by the Thermofluor assay (Boivin et al. 2013; Sutiono et al. 2018). The buffer used in the assay was 50 mM HEPES pH 7.5 with 5 mM EDTA, with or without 5 mM MgCl_2_.

#### Determination of pH optimum

A total of 20 μL of diluted enzyme (*Cn*L-KdpD with N-terminal His-tag or *Pp*D-KdpD) was added to a 96-well plate. A total of 180 μL reaction solution containing 5 mM of each substrate (L-KDP for *Cn*L-KdpD and D-KDP for *Pp*D-KdpD), 0.5 U/mL *Pp*KGSADH, 2 mM NAD^+^, 5 mM MgCl_2_, and 50 mM of buffer with corresponding pH (end concentration) was added. The reaction was monitored at 340 nm using Multiskan spectrophotometer to detect NADH formation. For pH 6 to 8, KPi buffer and for pH 7 to 9, Tris-HCl was used. Activities obtained were normalized to the highest value. Measurements were performed in triplicate. *Pp*KGSADH was confirmed to be active in the entire pH range tested (data not shown).

#### Effect of substrates and selected intermediates of the Weimberg pathway

D-Xylose and D-xylonate were used to check their inhibitory effects on *Pp*D-KdpD. L-Arabinose and L-arabinonate were used for *Cn*L-KdpD with a N-terminal fused His-tag. The experiments were performed as follows. 20 μL of diluted enzyme (*Pp*D-KdpD and *Cn*L-KdpD) was added to a 96-well plate along with 180 μL of reaction solution containing 5 mM of each substrate (L-KDP for *Cn*L-KdpD and D-KDP for *Pp*D-KdpD), 0.5 U/mL *Pp*KGSADH, 2 mM NAD^+^, 5 mM MgCl_2_, 50 mM HEPES pH 7.5, and increasing concentration of the respective inhibitors (end concentration). The formation of NADH was monitored at 340 nm using a Multiskan spectrophotometer. Measurements were performed in triplicate. The lack of inhibition of *Pp*KGSADH by the highest compound concentration tested (100 mM) was confirmed.

#### One-step conversion of high substrate loads

Regarding the *Cn*L-KdpD with a N-terminal His-tag and *Pp*D-KdpD, this was analyzed if high substrate load (L-KDP and D-KDP) concentrations would have an inhibitory effect. Each enzyme was used to convert each substrate in a reaction mix. The reaction mix contained 0.1 mg/mL enzyme, 5 mM MgCl_2_, 500 mM of respective substrate in 50 mM HEPES pH 7.5. The reaction mix was incubated in a thermoshaker (ThermoMixer C, Eppendorf, Germany) at 300 rpm and 25 °C. At certain times, 5 μL of the solution was removed from the reaction mix and diluted using 995 μL of ddH_2_O. A total of 500 μL of the diluted solution was transferred to a 10 KDa centrifugal filter (VWR, Germany), and the filter column was centrifuged at 12,000 ×*g* for 2 min. This step was used to stop the reaction by removing protein from the solution. The filtrate was used to analyze the formation of KGSA from either D-KDP or L-KDP by a previously established method (Beer et al. 2017). In brief, Metrosep A Supp 16–250 was used as a stationary phase, and 30 mM ammonium bicarbonate pH 10.4 was used as the mobile phase. The isocratic flow was set to 0.2 mL/min, and D-KDP, L-KDP, and KGSA were monitored by a UV detector at 210 nm. In this system, D-KDP and L-KDP showed the same retention time. KGSA eluted directly after D-KDP and L-KDP (Fig. S4 in the Supporting Information).

## Results

### Cloning, expression, and purification of the dehydratases

The position of a hexa-histidine tag (His-tag) sometimes has an effect on enzyme yield, stability, and activity. Therefore, *Cn*L-KdpD was cloned as an N-terminally as well as a C-terminally fused His-tag protein. After expression and purification, the protein yield of the N-Histag variant was almost 3 times higher (data not shown). In subsequent L-KdpDs, all variants were therefore cloned using N-terminally fused His-tags (Table 1). All variants were expressed using autoinduction media. A total of 20 to 50 mg of protein were able to be obtained from the 500 mL culture (except for *Ab*L-KdpD). To improve expression of *Ab*L-KdpD, a more enriched media (Terrific broth media) was used (Watanabe et al. 2006). From 500 mL culture, only 5 mg of this enzyme having lower protein purity were able to be obtained (Fig. S1). As a result, due to the difficulty with expression and the other L-KdpDs demonstrated having a similar magnitude of activity (Table 2), this enzyme would be of less interest with regard to application.

**Table 1:** List of L-KdpDs and D-KdpDs cloned in this study

Microorganism	Protein	NCBI accession number
*Caulobacter crescentus*	*Cc*D-KdpD1	WP_010918708.1
	*Cc*D-KdpD2	WP_012640070.1
*Paraburkholderia xenovorans*	*Px*D-KdpD	WP_011494434.1
*Pseudomonas putida* KT12440	*Pp*D-KdpD	WP_010953745.1
*Herbaspirillum seropedicae* Z67	*Hs*D-KdpD	WP_013235815.1
*Variovorax paradoxus* EPS	*Vp*D-KdpD	WP_013538688.1
*Cupriavidus necator* N-1	*Cn*D-KdpD	WP_011616492.1
*Azospirillum brasilense*	*Ab*L-KdpD	PDB: 3FKK
*Cupriavidus necator* N-1	*Cn*L-KdpD	WP_010809845.1
*Herbaspirillum seropedicae* Z67	*Hs*L-KdpD	WP_013233389.1
*Variovorax paradoxus* EPS	*Vp*L-KdpD	ADU35339.1

**Table 2:** Kinetic characterization of L-Kdp and D-Kdp dehydratases toward their natural isomer. All measurements were done in triplicate at 25 °C in 50 mM HEPES pH 7.5^a^

Substrate	Enzymes	k_cat_ (s^−1^)	K_M_ (mM)	k_cat_/K_m_ (mM^−1^ s^−1^)	Substrate preference^b^
L-KDP	*Ab*L-KdpD	13.6 ± 0.3	0.19 ± 0.02	71.3 ± 0.3	73
	*Cn*L-KdpD (Nhis)	23.4 ± 0.1	0.17 ± 0.00	137.2 ± 0.2	46
	*Cn*L-KdpD (Chis)	22.5 ± 0.2	0.16 ± 0.01	137.5 ± 0.2	80
	*Hs*L-KdpD	29.6 ± 0.2	0.18 ± 0.00	163.9 ± 0.2	63
	*Vp*L-KdpD	21.8 ± 0.2	0.15 ± 0.01	146.0 ± 0.2	62
D-KDP	*Cc*D-KdpD1	45.6 ± 0.3	0.25 ± 0.01	185.7 ± 0.2	65
	*Cc*D-KdpD2	55.8 ± 0.4	0.29 ± 0.01	190.6 ± 0.2	78
	*Px*D-KdpD	63.6 ± 0.4	0.28 ± 0.01	229.8 ± 0.2	62
	*Pp*D-KdpD	75.9 ± 0.6	0.35 ± 0.01	219.9 ± 0.2	69
	*Hs*D-KdpD	58.2 ± 0.4	0.17 ± 0.01	347.9 ± 0.2	65
	*Cn*D-KdpD	13.8 ± 0.2	0.23 ± 0.02	58.9 ± 0.4	35

^a^Error bars represent standard deviation from three replicates. Nonlinear regression of the enzyme activity as a function of substrate concentration is presented in Fig. S2

^b^The substrate preference was calculated as the ratio between the catalytic efficiency of the natural substrate and the non-preferred stereoisomer presented in Table S2

*Cc*D-KdpD1 was used as a template for analyzing which His-tag variant (N- or C-terminally fused His-tag) would be better with respect to activity and stability of D-KdpDs. Both His-tag variants of *Cc*D-KdpD1 were able to be expressed and purified, and similar protein yields were also obtained. However, the N-terminal variant precipitated rather quickly during enzyme characterization. With respect to all D-KdpDs, all variants therefore were cloned using C-terminally fused His-tags (Table 1). We could not obtain reasonable yield for *Vp*D-KdpD, therefore this variant was not pursued further.

### Kinetic characterization of the respective dehydratases

Regarding the enzymes purified, L-KdpD showed higher a preference for L-KDP than for D-KDP by at least 45-fold (Table 2). The activity (k_cat_) and K_M_-values of all L-KdpDs are also of the same magnitude as reported for the first L-KdpD from *Pseudomonas saccharophila* published in the late 1960’s (Stoolmiller and Abeles 1966). Regarding D-KdpDs, all enzymes exhibited a higher a preference for D-KDP over L-KDP by over 60-fold, except for *Cn*D-KdpD, which showed only a 35-fold higher preference. The activities of *Cc*D-KdpD1 and *Px*D-KdpD were considerably higher than reported in earlier research. The significant difference is presumably due to the fact that the authors in the previous study expressed *Cc*D-KdpD1 and *Px*D-KdpD with a N-terminally fused His-tag (Tai et al. 2016). The in vivo production of chemicals using *Cc*D-KdpD1 and *Px*D-KdpD from D-xylose has been demonstrated and no accumulation of D-KDP was reported. This could indicate that the enzyme introduced in *E. coli* did not bear any His-tag. Two enzymes from each class showing the highest activity and catalytic efficiency were then selected for further studies. *Cn*L-KdpD (Nhis) and *Hs*L-KdpD were chosen for L-KdpD and *Pp*D-KdpD and *Hs*KdpD for D-KdpD conversion.

### Effect of magnesium on activity, and the kinetic and thermodynamic stabilities of the most promising L-KdpDs and D-KdpDs

**Fig. 2:**
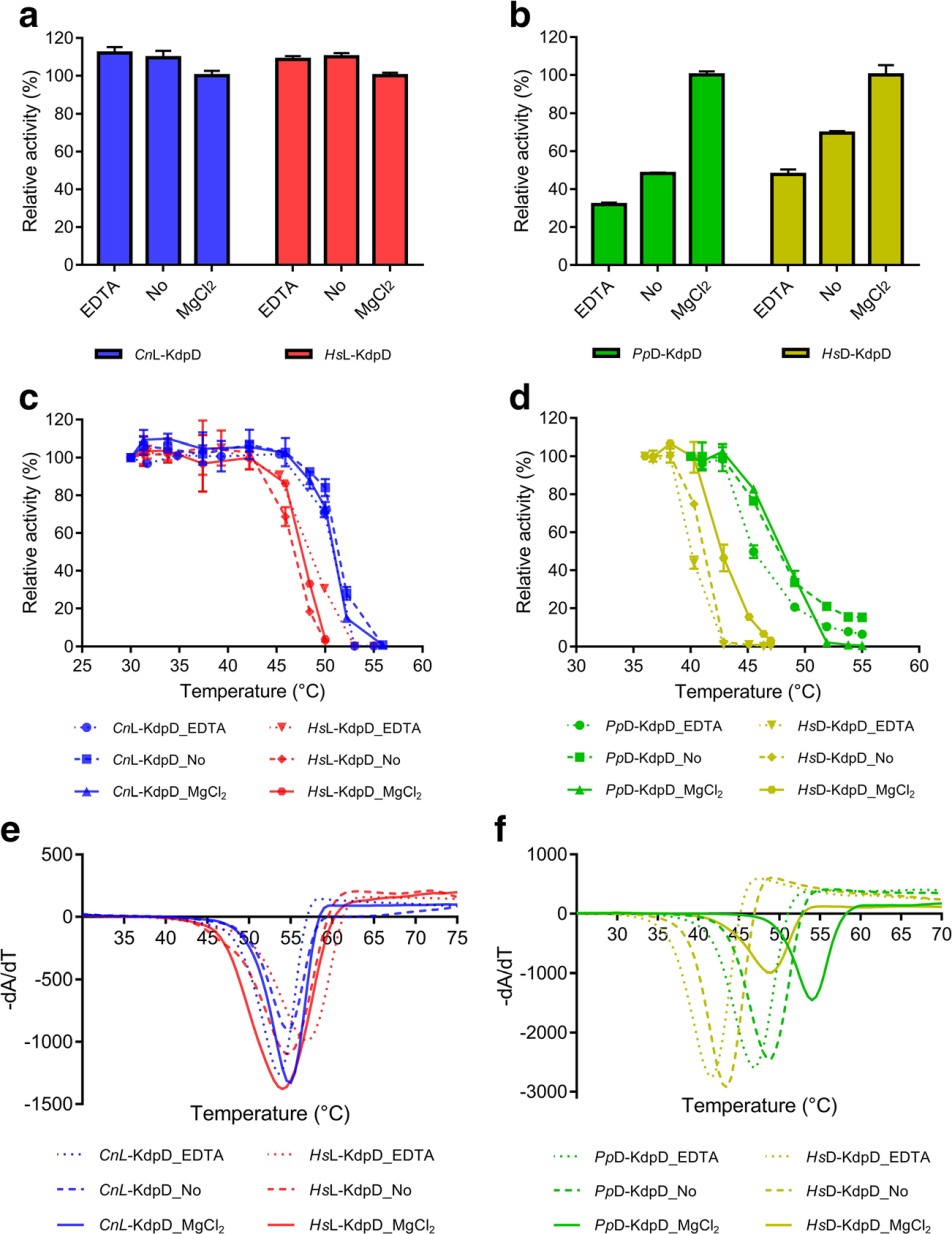
Influence of MgCl_2_ and EDTA on the activity, kinetic, and thermodynamic stabilities of L-KdpDs and D-KdpDs. L-KdpDs (**a**) and D-KdpDs (**b**) were pre-incubated with 5 mM EDTA (labeled with EDTA) prior to activity measurement using the coupled enzyme assay in the absence of MgCl_2_. In addition, activities were also determined without EDTA pre-incubation in the absence (labeled with “No”) and presence of 5 mM MgCl_2_. The kinetic stability of L-KdpDs (**c**) and D-KdpDs (**d**) was assayed as T_50_^1h^ (temperature at which 50% of kinetic activity is lost after 1 h of incubation). The thermodynamic stability of L-KdpDs (**e**) and D-KdpDs (**f**) was determined using Thermofluor and is represented as the melting temperature (T_m_). The effect of the addition of EDTA (5 mM), the absence of MgCl_2_, and the addition of MgCl_2_ (5 mM) in the enzyme solution was investigated for both enzymes. The numerical values are presented in Table S3

Magnesium has been reported as playing a role in D-KdpD from *Sulfolobus solfataricus* (*Ss*D-KdpD) (Brouns et al. 2006; Brouns et al. 2008). The cation is also observed in the crystal structure of *Ss*D-KdpD. However, *Ps*L-KdpD, which is the enzyme catalyzing the respective dehydration of the L-stereoisomer, was reported to be active without the addition of magnesium ions (Stoolmiller and Abeles 1966; Portsmouth et al. 1967). Magnesium ions have also been reported for the activity and stability of several other lyases, e.g., Fe-S dependent dehydratases and decarboxylases (De La Plaza et al. 2004; Andberg et al. 2016). This work studied the effect of magnesium ions on the most promising L-KdpDs and D-KdpDs. There was no apparent effect of magnesium on the activity of *Cn*L-KdpD and *Hs*L-KdpD (Fig. 2a). Nor did pretreatment of the enzymes with EDTA affect their activity. A different result was observed for *Pp*D-KdpD and *Hs*D-KdpD. When magnesium ions were not present during the activity measurement, a significant reduction of activity was observed (Fig. 2b), and the initial activity also seemed to be delayed (Fig. S3). Pretreatment with EDTA further decreased the initial activity.

Kinetic stability is also an important parameter in determining the applicability of an enzyme. In this study, T_50_^1h^ was used as an indicator of kinetic stability. This value can be used to reliably compare kinetic stability of enzymes given the same experimental conditions. *Cn*L-KdpD appeared to have higher kinetic stability (T_50_^1h^ of 53.2 °C) in comparison to *Hs*L-KdpD (T_50_^1h^ of 47.1 °C) (Fig. 2c). Again, it was shown that the absence of magnesium had barely any effect in regard to their kinetic stability. The presence of 5 mM metal chelator EDTA (> 250 equivalent) also did not decrease nor increase the kinetic stability of the two L-KdpDs. Regarding the two D-KdpDs, *Pp*D-KdpD revealed a T_50_^1h^ of 48.0 °C in the presence of magnesium compared to a T_50_^1h^ of 42.5 °C for *Hs*D-KdpD. The absence of magnesium did not significantly affect the kinetic stability of *Pp*D-KdpD. However, in the presence of 5 mM EDTA a slight decrease in kinetic stability (T_50_^1h^ lowered by 2.5 °C) was observed. The T_50_^1h^ of *Hs*D-KdpD was decreased by 1.3 °C and 2.4 °C in the absence of magnesium and presence of 5 mM EDTA, respectively.

In the present study, the thermodynamic stability is represented by the melting temperature (T_m_) and was determined by a thermal shift assay using Sypro orange dye. No apparent effect of magnesium in either of the representative L-KdpDs was found. The presence of 5 mM EDTA (>2500 equivalent) did not alter the T_m_ of *Cn*L-KdpD significantly, but it slightly increased the T_m_ of *Hs*L-KdpD (Fig. 2e). *Cn*L-KdpD and *Hs*L-KdpD appeared to have similar T_m_, although *Cn*L-Kdpd showed a higher T_50_^1h^. The stabilizing effect of magnesium was observed for the representative D-KdpDs. An addition of 5 mM magnesium increased the T_m_ of *Pp*D-KdpD and *Hs*D-KdpD by 5.5 and 5.1 °C, respectively. The presence of 5 mM EDTA (>2500 equivalent) decreased the T_m_ of both enzymes by about 2 °C, further supporting the effect of magnesium on their thermodynamic stability. Given that kinetic stability is a more relevant parameter in practical enzyme application in vivo and in vitro than thermodynamic stability, this parameter was used to further select the most promising enzyme variant from each dehydratase class. *Cn*L-KdpD and *Pp*D-KdpD were chosen for the determination of their pH profiles, the effect of selected intermediates of the Weimberg pathway, as well as suitability for high substrate conversion.

### Optimum pH for *Pp*D-KdpD and *Cn*L-KdpD

**Fig. 3:**
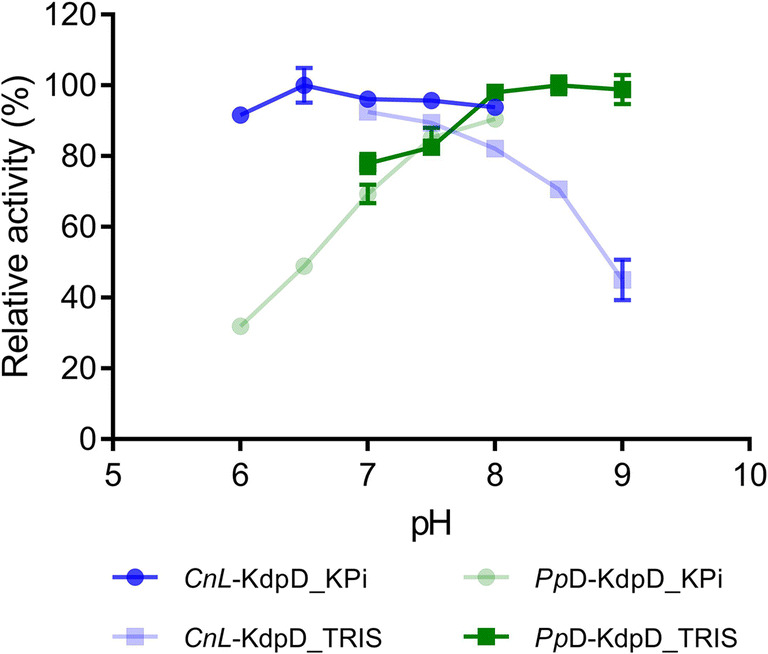
Optimum pH of *Cn*L-KDAD and *Pp*D-KdpD. *Cn*L-KDAD revealed an optimum pH of between 6.5 and 7 (darker blue), whereas *Pp*D-KdpD possessed an optimum pH of between 8 and 9 (darker green). Error bars represent the standard deviation of the three replicates

The pH profile of an enzyme is an important parameter for its application in vitro and in vivo. In particular, the pH of a reaction can be adjusted easily in a cell-free approach. Both enzymes, *Cn*L-KdpD and *Pp*D-KdpD, showed broad pH activity (Fig. 3). However, the main difference was that *Cn*L-KdpD showed higher activity under acidic conditions (optimum pH at 6.5 to 7) and showed significantly reduced activity (> 50% reduction) at pH 9. *Pp*D-KdpD, on the other hand, showed a preference for alkaline conditions (optimum pH at 8 to 9), and its activity was significantly reduced at pH 6 (70% reduction). There was no apparent effect on either enzyme activity by the buffer used, KPi or TRIS. A slightly acidic pH optimum was previously reported for *Ps*L-KdpD (Stoolmiller and Abeles 1966).

### Effect of upstream intermediates of the Weimberg pathways on *Cn*L-KdpD and *Pp*D-KdpD

In a multi-enzymatic reaction, intermediates can be accumulated depending on varying enzyme activities in a cascade reaction. Therefore, it would be important to determine whether the substrates or intermediates of the Weimberg pathway would have an inhibitory effect on *Cn*KdpD and *Pp*D-KdpD. L-arabinose and L-arabinonate were selected for the oxidative L-arabinose pathway and D-xylose and D-xylonate were selected for the oxidative D-xylose pathway. KGSA and α-KG were excluded in this study because the activities of *Cn*KdpD and *Pp*D-KdpD were determined by a coupled assay using *Pp*KGSADH as auxiliary enzyme to oxidize the formed KGSA to α-KG in the presence of NAD^+^. As for α-KG, not all modified pathways of D-xylose and L-arabinose will end or go via α-KG, e.g., toward 1,4-BDO formation (Fig. 1). Therefore, the effect of α-KG and other intermediates in the synthetic pathways would need to be determined later based on the desired target products.

**Fig. 4:**
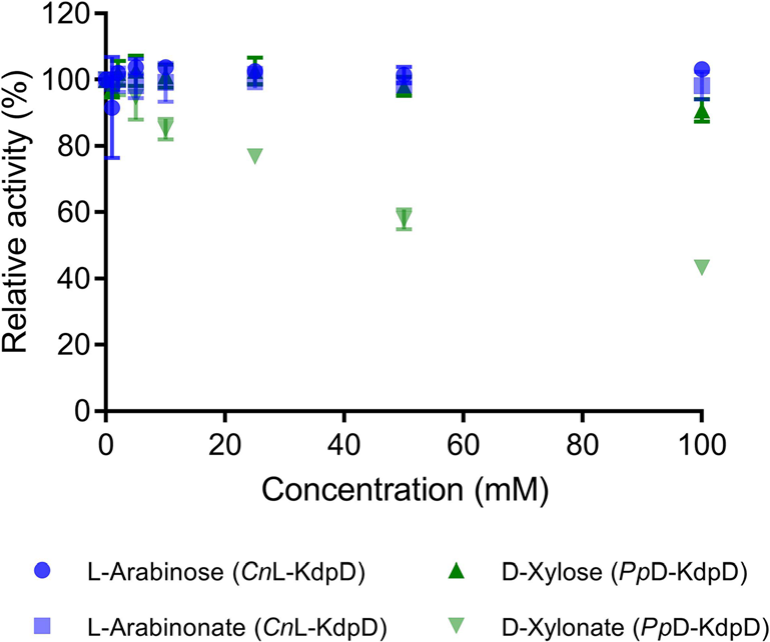
Effect of substrates and intermediates of the Weimberg pathway on the activity of *Cn*L-KdpD and *Pp*D-KdpD. There was no inhibitory effect of D-xylose up to 100 mM on the activity of *Pp*D-KdpD, as well as L-arabinose and L-arabinonate on the activity of *Cn*L-KdpD. D-xylonate inhibited *Pp*D-KdpD activity at an I_50_-value of 75 mM

*Cn*LKdpD demonstrated no inhibitory effects in the presence of L-arabinose and L-arabinonate up to 100 mM (Fig. 4). These characteristics would make *Cn*LKdpD a suitable enzyme for application in the non-phosphorylative oxidative conversion of L-arabinose (McClintock et al. 2017). As for *Pp*D-KdpD, the enzyme was inhibited in the presence of D-xylonate with an I_50_ value (the concentration of a compound that gives 50% inhibition to the initial activity of an enzyme) of 75 mM, whereas the *Pp*D-KdpD was not inhibited by D-xylose (Fig. 4). *Pp*KGSADH, the auxiliary enzyme in the coupled assay, was also confirmed not to be inhibited by D-xylonate. Therefore, the inhibition effect shown was only for *Pp*D-KdpD.

### One-step conversion of L-KDP and D-KDP to KGSA using *Cn*L-KdpD and *Pp*KdpD

**Fig. 5:**
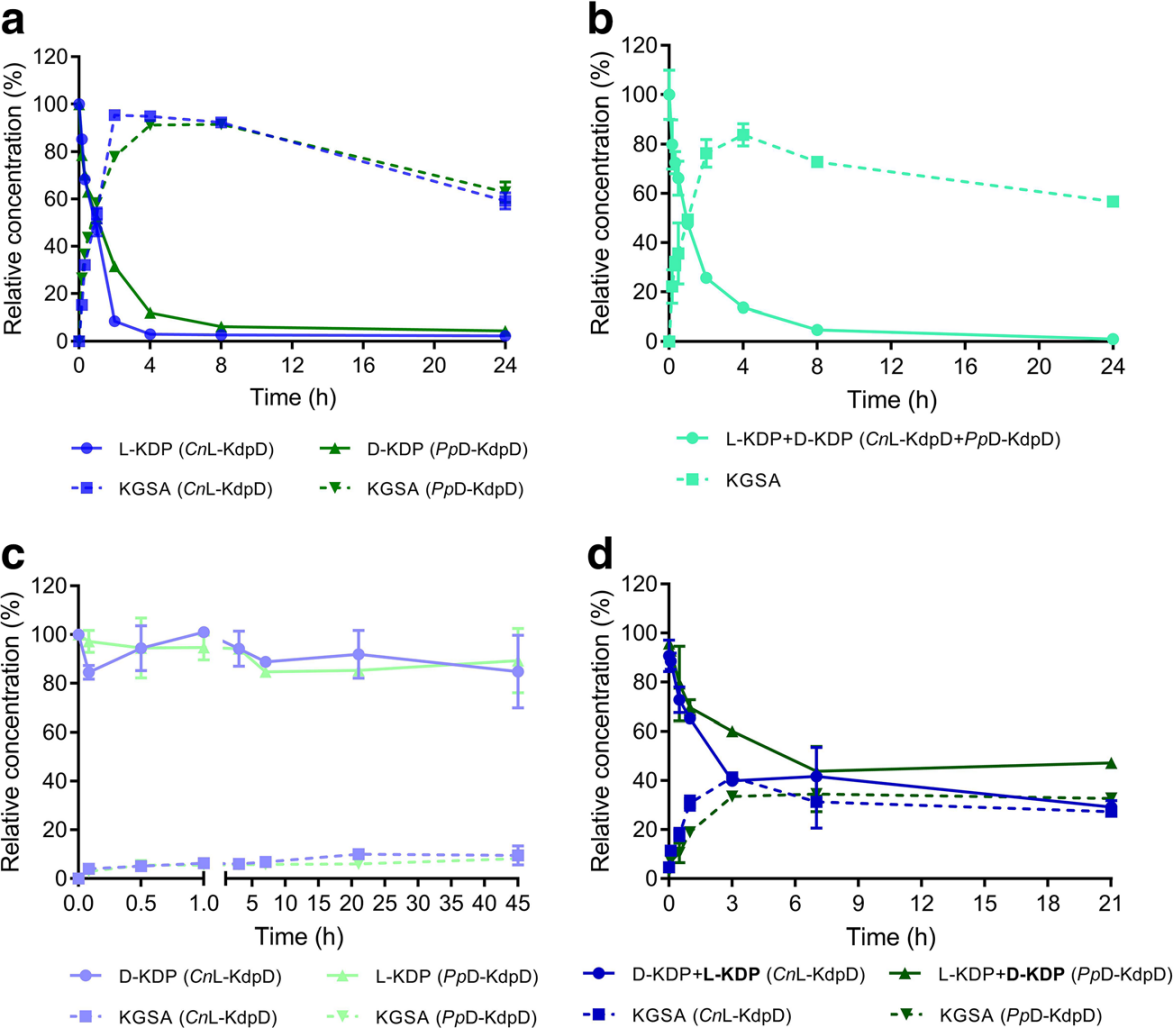
Time-dependent conversion of L-KDP and D-KDP to KGSA using *Cn*L-KdpD and *Pp*D-KdpD. A total of 0.1 mg/mL of each enzyme was used to convert 500 mM of the respective KDP (**a**,**c**). A combination of *Cn*L-KdpD and *Pp*D-KdpD (0.05 mg/mL each) was used to convert 500 mM of a racemic mixture of D,L-KDP (**b**). After each enzyme was used to convert the non-preferred isomer for 21 h (in panel c), 100 μL of each solution was transferred to 100 μL with 500 mM of the respective natural isomer (labeled with bold) to see if both enzymes were still able to convert their natural substrate (**d**). All experiments were performed in HEPES pH 7.5 at 25 °C

A high substrate load is often desirable for the biotransformation of chemicals. To determine the feasibility of both enzymes respecting high substrate load conversion, each enzyme was used to transform 500 mM D-KDP and L-KDP. A previously established HPLC analysis was utilized for this experiment (Beer et al. 2017). For the conversion of D-KDP, *Pp*D-KdpD was able to reach 90% conversion to KGSA after 4 h. For L-KDP, *Cn*L-KdpD reached > 95% conversion after only 2 h (Fig. 5a). In the first 10 min, *Pp*D-KdpD showed a higher turnover rate than *Cn*L-KdpD, but the conversion of *Pp*D-KdpD slowed over time. When the concentration of KGSA reached 300 mM, the activity of *Pp*D-KdpD was only 17 U/mg, representing only 12% of its initial activity (in the first 10 min). As for *Cn*L-KdpD, its activity was 38 U/mg in the presence of 300 mM KGSA, representing 45% of its initial activity. A combination of both enzymes was also able to convert a 500 mM racemic mixture of D,L-KDP (250 mM of each isomer). In this experimental set-up, > 80% conversion was observed after 4 h (Fig. 4b). The concentrations of KGSA decreased after 8 h, and a further decrease after 24 h of incubation was noticed in both set-ups (Fig. 4a and b). The degradation of KGSA was likely due to interaction of its terminal aldehyde group with amino acids on the protein surface (Robert and Penaranda 1954; Uchida 2003; Grimsrud et al. 2008). This is further supported by the yellow color of the reaction solutions observed after 24 h of reaction, which is indicative of a typical aldehyde protein interaction.

During the kinetic characterization, it was shown that both enzymes were also reactive toward the other non-preferred stereoisomer (*Pp*D-KdpD toward L-KDP and *Cn*L-KdpD toward D-KDP) although at a much lower efficiency. A similar finding was previously reported for the D-KdpD from *Herbaspirillum huttiense* and L-KdpD from *Paraburkholderia mimosarum* (Watanabe et al. 2019). The application of only one enzyme for the conversion of both stereoisomers would be of interest for practical applications such as the conversion of mixture of sugars, e.g., L-arabinose and D-xylose, into certain chemicals. Therefore, in order to confirm their promiscuity, each enzyme was used to convert 500 mM of the non-preferred stereoisomer. As shown in Fig. 4c, both enzymes were active toward the other non-preferred stereoisomer. However, the reaction appeared to have halted rather quickly. After 21 and 45 h of reaction, only a slightly higher formation of KGSA was observed for both enzymes. HPLC analysis indicated that the product formed was indeed KGSA because the same retention time was observed (Fig. S4). When the solution of each enzyme after 21 h reaction with the other non-preferred isomer (Fig. 5c) was transferred to a new solution containing 500 mM of their natural isomer, the formation of KGSA was immediately initiated (Fig. 5d). This indicates that the presence of high concentration of the non-preferred isomer did not have any negative effect for both enzymes. *Cn*L-KdpD reached 40% of the theoretical yield of KGSA (80% if calculated from L-KDP only), whereas *Pp*D-KdpD reached 33% of the theoretical yield of KGSA (66% from D-KdpD) after 3 h.

## Discussion

D-xylose and L-arabinose are the two most abundant pentose sugars in hemicellulose. Utilization of these sugars to produce platform chemicals would allow valorization of lignocellulose biomass. The enzymatic cascade reactions of the Weimberg pathways have been used to transform these pentose sugars into several chemicals, e.g., BDO, α-KG, succinate, glutamate, and mesaconate (Fig. 1). These chemicals are still industrially produced or derived from petrochemicals. Enzymes for the key steps of the Weimberg pathway, specifically L-KdpD and D-KdpD, have only been partially characterized with major focus on their kinetic properties. These specific enzymes catalyze the dehydration of L-KDP and D-KDP to KGSA.

In this study, several L-KdpDs and D-KdpDs were cloned and heterologously expressed in *E. coli*. For the *Cn*L-KdpD, the N-terminally fused His-tag resulted in higher protein yields compared to the C-histag variant. For D-KdpD, however, the C-histag was the better variant. The fact of the N-terminally fused variant of *Cc*D-KdpD1 quickly precipitating and the higher activity of *Cc*D-KdpD2, which is a homologous enzyme of *Cc*KdpD1 (58 amino acids shorter at the N-terminus, Fig. S1) as compared to *Cc*D-KdpD1, could suggest that the N-terminus for D-KdpD is important to enzyme activity and/or stability, and that the addition of further amino acids N-terminally would impede either. However, in a recent study, N-terminally fused His-tag of *Cc*KdpD2 (bearing additional 20 amino acids in N-terminus in comparison to C-histag of *Cc*KdpD2 in this study) showed a magnitude of activity similar to *Cc*KdpD2 (Shen et al. 2020). Further analysis using BLAST searches in the NCBI and UniProt database suggest that the previously cloned and characterized *Cc*D-KdpD1 might be cloned with the wrong start codon (Tai et al. 2016), since most of the homologs do not show the 58 amino acids extension at the N-terminus. The amino acid downstream of the current start codon (the 59th amino acid encoded by GTG) is a second possible start site. Therefore, future studies have to be completed in order to confirm whether the truncated version is more stable and/or active and to decide whether the length of the additional amino acids is also an important factor.

All L-KdpDs revealed a high catalytic efficiency (> 100-fold) with L-KDP, whereas all D-KdpDs showed high efficiency (> 150-fold) with D-KDP. The catalytic efficiency of the KdpDs is an important parameter for in vitro and in vivo pathway design. In particular, the catalytic efficiency for D-KdpD measured in vitro has been shown to have a strong correlation with enzyme efficiency in vivo (Tai et al. 2016). In the previous study, it was shown that the *E. coli* mutant expressing *Px*D-KdpD produced BDO faster than the *E. coli* mutant expressing *Cc*D-KdpD1 (Tai et al. 2016). The authors (as well as this study) have demonstrated a higher catalytic efficiency for *Px*D-KdpD than for *Cc*D-KdpD1 (Table 2). Furthermore, in the recent study combining enzymatic and modeling approach, the authors were able to predict and simulate the activity of all five Weimberg enzymes, including *Cc*D-KdpD2 in cell-free extracts based on their kinetic model (Shen et al. 2020).

A study on the effect of magnesium on activity, kinetic, and thermodynamic stability revealed that this cation showed effects on members of the D-KdpD class, while no apparent effects were observed on the enzymes of the L-KdpD class. The presence of magnesium increased the activity and kinetic (T_50_^1h^) and thermodynamic (T_m_) stabilities of *Pp*D-KdpD and *Hs*D-KdpD. No significant difference was observed when comparing the thermodynamic stability of *Cn*L-KdpD and *Hs*L-KdpD, although the kinetic stability suggested otherwise. Apparently, the relationships between the kinetic and thermodynamic stabilities were not able to be observed for L-KdpD, which stands in contrast to our previous studies of two α-keto acid decarboxylases (Sutiono et al. 2018; Sutiono et al. 2019).

The detailed characterization with respect to pH profiles revealed an optimum pH at 6.5 to 7 for *Cn*L-KdpD, whereas *Pp*D-KdpD showed the highest activity at pH 8 to 9. Together with the differing effect of magnesium toward L- and D-KdpD, these results suggest that L-KdpD and D-KdpD may adopt different catalytic mechanisms for performing the dehydration at position C4 and C5 of L-KDP and D-KDP, respectively. This outcome could also imply that different amino acid residues are acting during the catalysis, since the pH level would determine the protonation states of charged amino acids. This hypothesis is further supported by the fact that, from the automated gene annotation in these two enzyme classes, although they catalyze similar reactions, they belong to different enzyme families. All D-KdpDs are annotated as members of the fumarylacetoacetate hydrolase (FAH) family, whereas all L-KdpDs are annotated as members of the dihydrodipicolinate synthase (DHDPS) family. The relative similarity of each dehydratase in their corresponding family is presented in Fig. S5.

The effects of upstream substrates and intermediates in the Weimberg pathway on each enzyme were also studied. L-arabinose and L-arabinonate up to 100 mM did not show any inhibitory effect on *Cn*L-KdpD. Nor did the same concentration of D-xylose inhibit the activity of *Pp*-KdpD. However, D-xylonate showed a significant inhibition of *Pp*-KdpD at an I_50_-value of 75 mM. This concentration seems to be quite high (the higher the I_50_, the less significant the inhibitory effect). However, due to the low activity of D-xylonate dehydratase (Fig. 1), D-xylonate could accumulate to this relevant range. The [Fe-S]-dependent sugar acid dehydratase has been reported as being one of the major bottlenecks in in vitro and in vivo utilization of the Weimberg pathway (Salusjärvi et al. 2017; Boer et al. 2019; Shen et al. 2020). Therefore, a more active D-xylonate dehydratase would be needed to avoid an accumulation of D-xylonate that would inhibit *Pp*D-KdpD. In addition, due to the high k_cat_ value exhibited by *Pp*D-KdpD (75 s^−1^), it would be unlikely for this enzyme to be the bottleneck in the non-phosphorylative oxidative conversion of D-xylose (Fig. 1). A stronger inhibition effect of D-xylonate was reported for *Cc*D-KdpD2 (Shen et al. 2020).

In the one-step conversion, *Cn*L-KdpD was able to convert 500 mM of L-KDP to KGSA, reaching > 95% yield after only 2 h. When the same amount of *Pp*D-KdpD (higher initial activity) was used to convert D-KDP, the enzyme needed 4 h to reach > 90% yield. This indicates that the KGSA formed has a higher inhibitory effect on *Pp*D-KdpD than on *Cn*L-KdpD. The combination of both enzymes can convert 500 mM of a racemic mixture of D,L-KDP, reaching 83% yield after 4 h. An initial activity test of both enzymes suggests that each enzyme could convert the non-preferred stereoisomer of its natural substrate (Table S2). However, time-based experiments of the non-preferred isomer (Fig. 4c) reveal that both enzymes were only able to convert < 5%, even after 45 h of reaction. This could indicate that the non-preferred isomers inhibit each of the two enzymes. Another, more plausible, explanation is that most (if not all) of the KGSA was formed due to contamination with the natural stereoisomer. An impurity could be derived from the initial substrates used, e.g., aldoses or an unwanted epimerization at C4 which could occur during preparation of the D- and L-KDP. Further experiments, in which the non-preferred stereoisomer was titrated to each enzyme reaction, demonstrated that a greater substrate addition leads to a greater formation of KGSA (Fig. S6). This result suggests that each enzyme is somewhat more specific to the respective stereoisomer than appeared to be the case from the initial activity measurement (Table S2) (Stoolmiller and Abeles 1966). Apparently, the initial activity measurements are not enough to determine the promiscuity of these enzyme classes.

In conclusion, we have cloned, expressed, and characterized a number of L-KdpDs and D-KdpDs. On the basis of catalytic efficiency, activity, and kinetic stability, we demonstrated that *Cn*L-KdpD and *Pp*D-KdpD are the most promising variants from each enzyme class. Both enzymes possess a broad pH range. However, although *Cn*L-KdpD revealed the highest activity at a slightly acidic pH, *Pp*D-KdpD was more active at an alkaline pH. Both enzymes are suitable for high-substrate load conversion.

## Electronic supplementary material

ESM 1 (PDF 611 kb)
