# Supplementary material for: Characterization of highly active 2-keto-3-deoxy-L-arabinonate and 2-keto-3-deoxy-D-xylonate dehydratases in terms of the biotransformation of hemicellulose sugars to chemicals
Source: Appl Microbiol Biotechnol. 2020 Jun 21;104(16):7023–35. doi: 10.1007/s00253-020-10742-5 (PMC7374468; doi:10.1007/s00253-020-10742-5)
Supplement: Supplementary file 1 — (PDF 611 kb) [file 253_2020_10742_MOESM1_ESM.pdf]

Supplementary Materials

Applied Microbiology and Biotechnology

Characterization of highly active 2-keto-3-deoxy-L-arabinonate and 2-keto-3-deoxy-D-xylonate dehydratases in terms of the biotransformation of hemicellulose sugars to chemicals

Samuel Sutiono[a], Bettina Siebers[b], Volker Sieber[a,c,d,e*]

[a] Chair of Chemistry of Biogenic Resources, Campus Straubing for Biotechnology and Sustainability, Technical University of Munich, Schulgasse 16, 94315 Straubing, Germany

[b] Molecular Enzyme Technology and Biochemistry (MEB), Environmental Microbiology and Biotechnology (EMB), Centre for Water and Environmental Research (CWE), University of Duisburg-Essen, Universitätsstraße 5, 45117 Essen, Germany

[c] Catalytic Research Center, Technical University of Munich, Ernst-Otto-Fischer-Straße 1, 85748 Garching, Germany

[d] Straubing Branch BioCat, Fraunhofer IGB, Schulgasse 11a, 94315 Straubing, Germany

[e] School of Chemistry and Molecular Biosciences, The University of Queensland, 68 Copper Road, St. Lucia 4072, Australia

*Email: sieber@tum.de; ORCID: 0000-0001-5458-9330; Tel: +49 (0) 9421 187-300; Fax: +49 (0) 9421 187-310

Table S1. List of primers and restriction enzymes used to clone L-KdpDs and D-KdpDs in this study*

Protein	NCBI Accession number	Restriction enzymes	Primers (5´to 3´)
*Cc*D-KdpD1	WP_010918708.1	*Nde*I, *Xho*I	Ordered as an optimized gene for *E. coli* expression<br>(GenBank Accession number: MT550669)
*Cc*D-KdpD2	WP_012640070.1	*Nde*I, *Hind*III	Fwd: CAGCAGCATATGGGCGTGAGTGAATTCCTGCCGGAAGATTG<br>Rev: CAGCAGAAGCTTGAGGAGGCCGCGGCCGGC
*Px*D-KdpD	WP_011494434.1	*Nde*I, *Xho*I	Ordered as an optimized gene for *E. coli* expression<br>(GenBank Accession number: MT550670)
*Pp*D-KdpD	WP_010953745.1	*Nde*I, *Xho*I	Fwd: CAGCAGCATATGACTGATCGAACGAACGCCAATC<br>Rev: CAGCAGCTCGAGAAGCGGGCGGGCGGTGG
*Hs*D-KdpD	WP_013235815.1	*Nde*I, *Xho*I	Fwd: CAGCAGCATATGGCACACACCTTTTCGCTCCAGGCGC<br>Rev: CAGCAGCTCGAGAATGAGTTTGCGGTCGGCCAGATTTTTGAACAGCGC
*Vp*D-KdpD	WP_013538688.1	*Nde*I, *Eco*RI	Fwd: CAGCAGCATATGGCCCTGAACCTCTCGCCCGCGAC<br>Rev: CAGCAGGAATTCCAACAGGCCGCGCGCGCCCAGGTTG
*Cn*D-KdpD	WP_011616492.1	*Nde*I, *Xho*I	Fwd: CAGCAGCATATGGCATTGCCGCTCACGCTCAGCG<br>Rev: CAGCAGCTCGAGGAAAGTGGTGTGGCCACGTGCGGCC
*Ab*L-KdpD	PDB: 3FKK	*Nde*I, *Xho*I	Ordered as an optimized gene for *E. coli* expression<br>(GenBank Accession number: MT550671)
*Cn*L-KdpD	WP_010809845.1	*Nde*I, *Xho*I	Fwd: CAGCAGCATATGACCCGCACCCCATCCC<br>Rev1: CAGCAGCTCGAG**TTATCA**GCGGGCCCAGCGCAG<br>Rev2: CAGCAGCTCGAGGCGGGCCCAGCGCAG
*Hs*L-KdpD	WP_013233389.1	*Nde*I, *Xho*I	Fwd: CAGCAGCATATGAGCACCCCTCTCTACCGCGGCGTATTCCC<br>Rev: CAGCAGCTCGAG**TTA**CCTGCCCCAGCTCAGCACCAGCGGATC
*Vp*L-KdpD	ADU35339.1	*Nde*I, *Xho*I	Fwd: CAGCAGCATATGCCCAAGCCCATGAACCACAACCTCTCC<br>Rev: CAGCAGCTCGAG**TTA**CTTCCCCCAGCGCAGCACCAGCGGG

* Restriction site in the primers is underlined. Stop codon is highlighted bold.

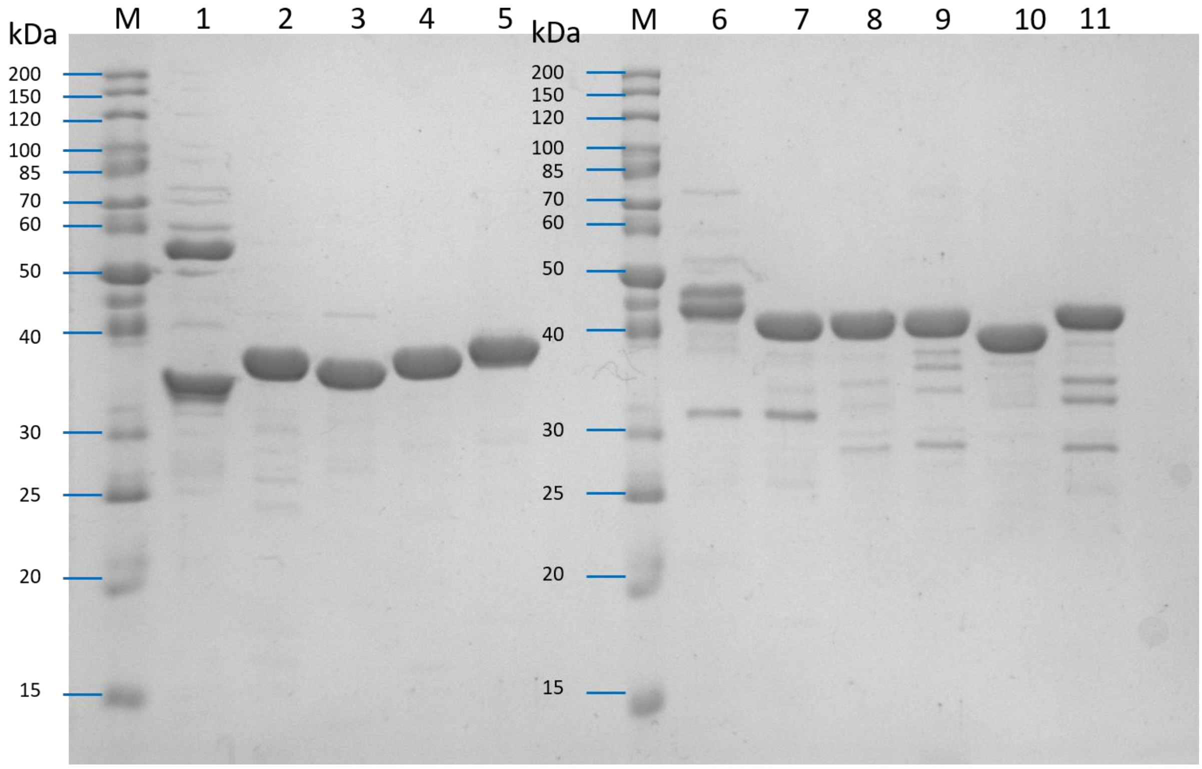

Figure S1. SDS-PAGE of all L-KdpDs (left) and D-KdpDs (right) after purification and desalting. From left to right: Marker, *Ab*L-KdpD, *Cn*L-KdpD with N-terminal His-tag, *Cn*-L-KdpD with C-terminal His-tag, *Hs*L-KdpD, *Vp*L-KdpD, Marker, *Cc*D-KdpD1, *Cc*D-KdpD2, *Px*D-KdpD, *Pp*D-KdpD, *Hs*D-KdpD, *Cn*D-KdpD.

Table 2. Apparent (APP) kinetic characterization of L-Kdp and D-Kdp dehydratases toward their non-preferred stereoisomer. All measurements were performed in triplicate at 25 °C, in 50 mM HEPES pH 7.5.*

Substrate	Enzymes	k_{cat}^{APP} (s^{-1})	K_M^{APP} (mM)	k_{cat}/K_m ($mM^{-1}s^{-1}$)
D-KDP	*Ab*L-KdpD	6.65 ± 0.10	6.81 ± 0.35	0.98 ± 0.23
	*Cn*L-KdpD (Nhis)	12.04 ± 0.16	4.08 ± 0.21	2.95 ± 0.23
	*Cn*L-KdpD (Chis)	11.01 ± 0.08	6.40 ± 0.17	1.72 ± 0.16
	*Hs*L-KdpD	11.38 ± 0.06	4.41 ± 0.09	2.58 ± 0.14
	*Vp*L-KdpD	11.78 ± 0.19	4.99 ± 0.30	2.36 ± 0.25
L-KDP	*Cc*D-KdpD1	7.99 ± 0.08	2.81 ± 0.12	2.84 ± 0.21
	*Cc*D-KdpD2	9.67 ± 0.08	3.96 ± 0.13	2.44 ± 0.18
	*Px*D-KdpD	18.73 ± 0.15	5.08 ± 0.17	3.69 ± 0.18
	*Pp*D-KdpD	19.64 ± 0.17	6.12 ± 0.21	3.21 ± 0.19
	*Hs*D-KdpD	16.92 ± 0.21	3.18 ± 0.18	5.32 ± 0.23
	*Cn*D-KdpD	4.24 ± 0.07	2.49 ± 0.20	1.70 ± 0.28

* Error bars represent standard deviation from three replicates. Nonlinear regression of the enzyme activity as a function of substrate concentration is presented in Figure S2.

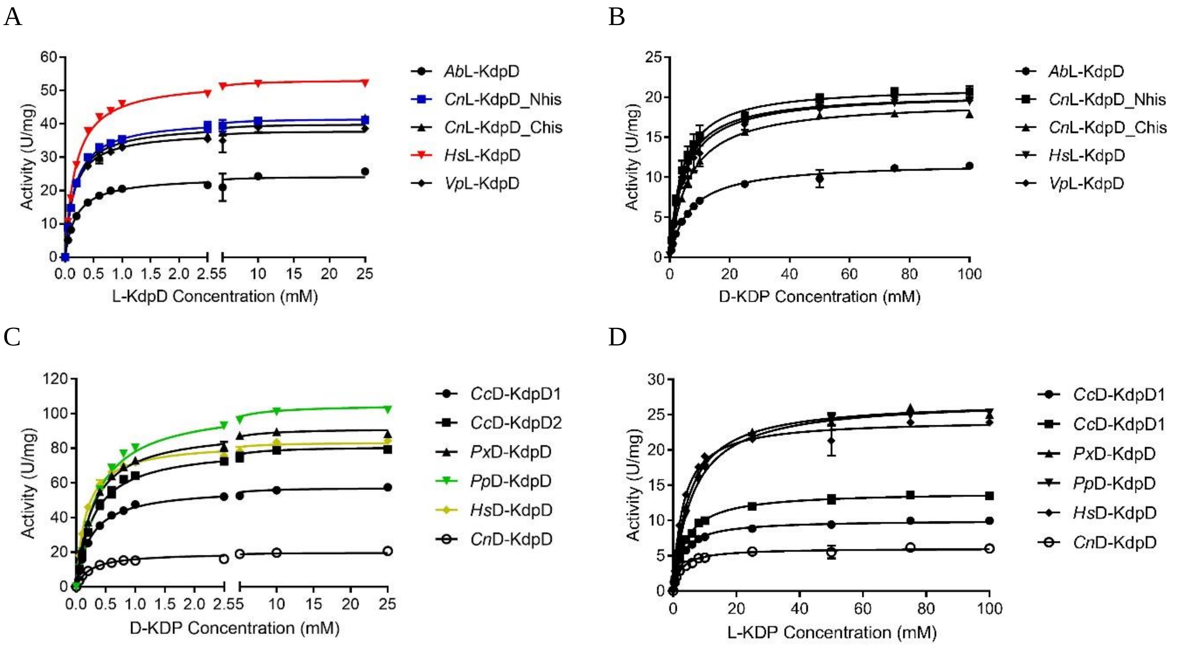

Figure S2. Michaelis-Menten kinetics of all dehydratases used in this study. Error bars represent standar deviation from three independent replicates. Activity was determined by a coupled-assay with *Pp*KGSADH. One unit is defined as the amount of enzyme to convert 1 µmol of NAD^+ per minute. The k_{cat}-values presented in Table 1 were calculated based on the specific activity (U/mg) obtained from this graph divided by the enzyme used. All experiments were performed in HEPES 50 mM pH 7.5, 25 °C

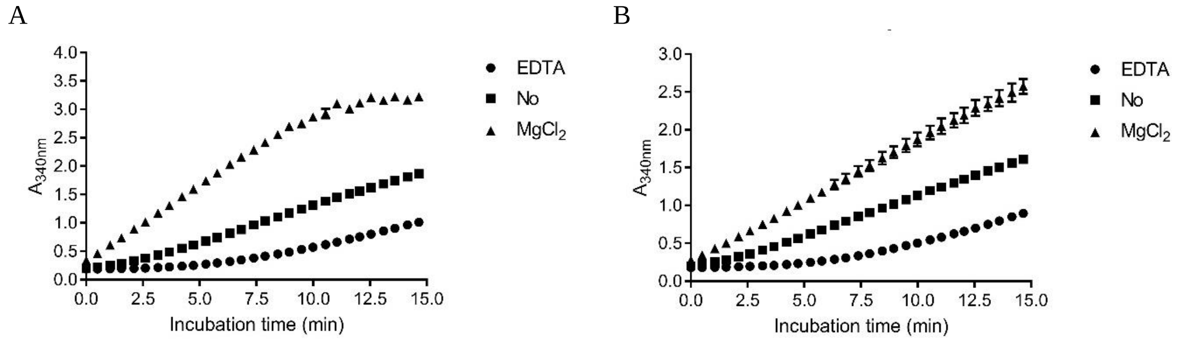

Figure S3. Effect of pre-incubation with EDTA as well as absence, and presence of $MgCl_2$ on the initial activity of *Pp*D-KdpD (A) and *Hs*D-KdpD (B). There is apparent delay in the initial activity of *Pp*D-KdpD and *Hs*D-KdpD after pretreatment with EDTA (no magensium presents in the assay) and when magnesium was absent during the assay. Thus, for the initial activity determination, the enzymatic activity (EDTA and No) was calculated from minute 8 to 12. Error bars represent standard deviation for three replicates.

Table S3. Effect of the presence of EDTA, absence of Mg^{2+}, and presence of Mg^{2+} on the kinetic (T_{50}^{1h}) and thermodynamic (T_m) stabilities of respective L-KdpDs and D-KdpDs as presented in Figure 1 D-F

Enzyme	T_{50}^{1h} (°C)			T_m (°C)		
	EDTA	No	Mg	EDTA	No	Mg
*Cn*L-KdpD	53.2 ± 0.1	53.2 ± 0.0	53.1 ± 0.0	53.5 ± 0.0	54.5 ± 0.0	55.0 ± 0.0
*Hs*L-KdpD	48.0 ± 0.1	47.3 ± 0.1	47.1 ± 0.1	57.0 ± 0.0	54.5 ± 0.0	54.0 ± 0.0
*Pp*D-KdpD	45.5 ± 0.4	47.7 ± 0.0	48.0 ± 0.2	46.8 ± 0.3	48.8 ± 0.3	54.3 ± 0.6
*Hs*D-KdpD	40.1 ± 0.1	41.2 ± 0.0	42.5 ± 0.7	41.3 ± 0.3	43.7 ± 0.3	48.8 ± 0.3

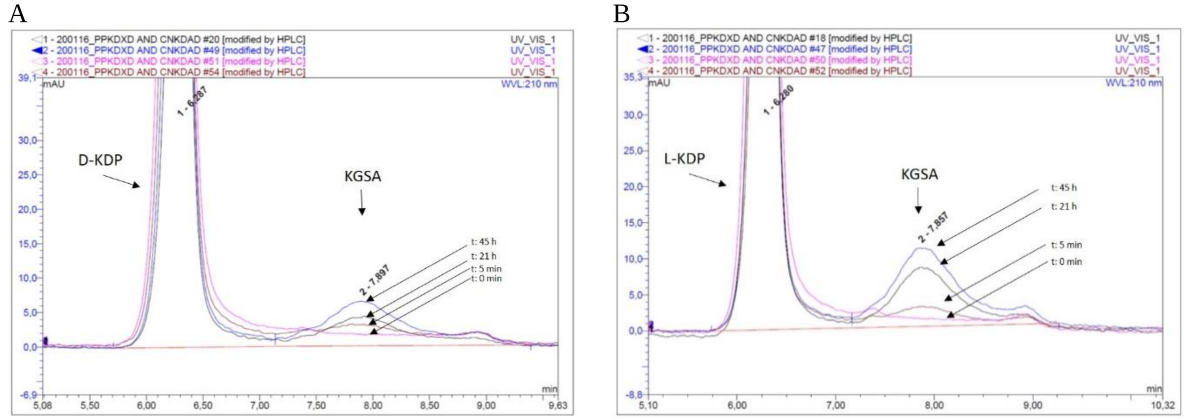

Figure S4. Chromatogram of the conversion of D-KDP (non-preferred stereoisomer) to KGSA by *Cn*L-KdpD (A) and L-KDP (non-preferred stereoisomer) to KGSA by *Pp*D-KdpD (B). D-KDP and L-KDP showed the same retention time. The conversion of D-KDP to KGSA by *Cn*L-KdpD appeared to be severely slow in comparison to the initial activity presented in Table 2. Similar results were observed for the conversion of L-KDP to KGSA by *Pp*D-KdpD. Retention times of D- or L-KDP are the same at 6.28 min while retention time of KGSA is at 7.86 min.

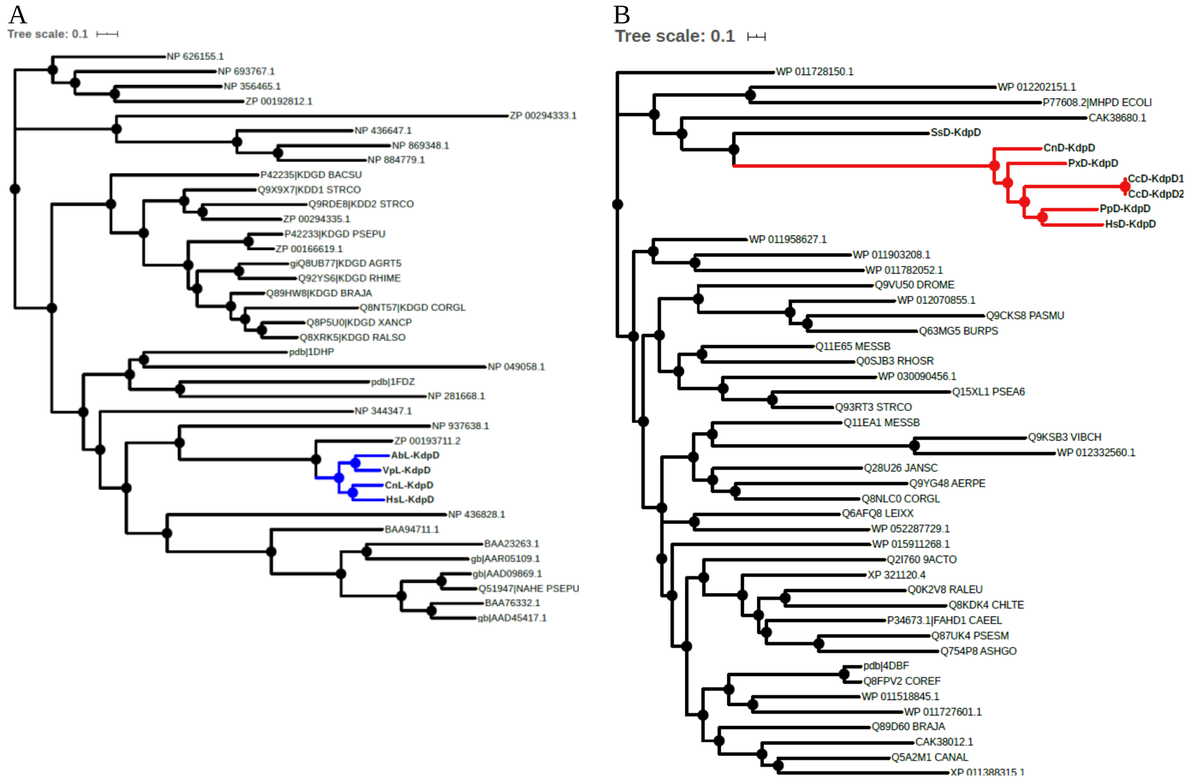

Figure S5. Phylogenetic tree of L-KdpDs (blue clades) in relation to other enzymes in dihydrodipicolinate synthase super family (DHDPS) (A) and D-KdpDs (red clades) in relation to enzymes in fumarylacetocatate hydrolase super family (FAH) (B). The sequences of other members of each family are retrieved from the Conserved Domain Database (Marchler-Bauer et al. 2016). Sequence alignment and phylogenetic tree construction were performed using MEGA-X software (Kumar et al. 2018). Sequence alignment was performed using the MUSCLE algorithm and the phylogenetic tree was developed using Maximum Likelihood algorithm embedded in MEGA-X using default parameters (Jones et al. 1992; Edgar 2004). The trees in Newick format were visualized using iTOL (Interactive Tree Of Life) (Letunic and Bork 2019).

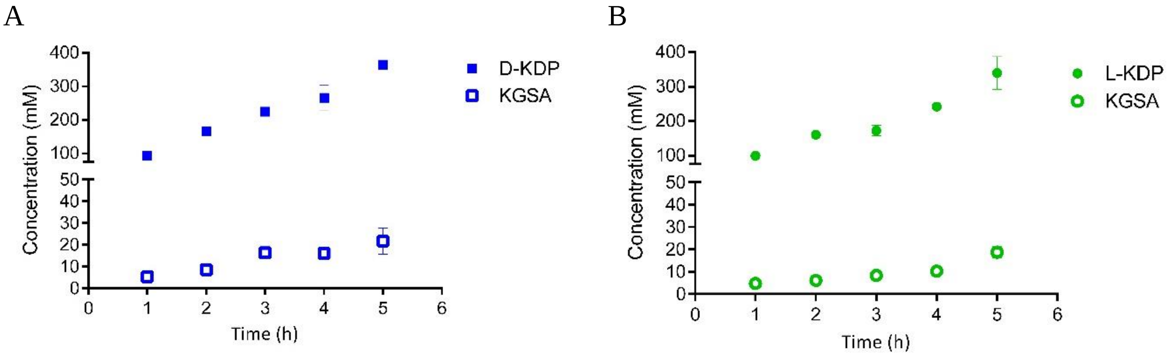

Figure S6. Effect of titration of D-KDP to *Cn*L-KdpD (A) and L-KDP to *Pp*D-KdpD (B). After each addition of the substrates, both solutions were incubated for 1 h, prior to analysis of KGSA formation via HPLC. Higher formation of KGSA was observed when more substrate was added for both enzyme indicating the KGSA formed could be from the impurity, i.e. the other KDP isomer.

References

Edgar RC (2004) MUSCLE: a multiple sequence alignment method with reduced time and space complexity. BMC Bioinformatics 5:113 . https://doi.org/10.1186/1471-2105-5-113

Jones DT, Taylor WR, Thornton JM (1992) The rapid generation of mutation data matrices from protein sequences. Bioinformatics 8:275–282 . https://doi.org/10.1093/bioinformatics/8.3.275

Kumar S, Stecher G, Li M, Knyaz C, Tamura K (2018) MEGA X: Molecular Evolutionary Genetics Analysis across Computing Platforms. Mol Biol Evol 35:1547–1549 . https://doi.org/10.1093/molbev/msy096

Letunic I, Bork P (2019) Interactive Tree Of Life (iTOL) v4: recent updates and new developments. Nucleic Acids Res 47:W256–W259 . https://doi.org/10.1093/nar/gkz239

Marchler-Bauer A, Bo Y, Han L, He J, Lanczycki CJ, Lu S, Chitsaz F, Derbyshire MK, Geer RC, Gonzales NR, Gwadz M, Hurwitz DI, Lu F, Marchler GH, Song JS, Thanki N, Wang Z, Yamashita RA, Zhang D, Zheng C, Geer LY, Bryant SH (2016) CDD/SPARCLE: functional classification of proteins via subfamily domain architectures. Nucleic Acids Res 45:D200–D203 . https://doi.org/10.1093/nar/gkw1129
